# Memantine ameliorates oxaliplatin-induced neurotoxicity via mitochondrial protection

**DOI:** 10.1080/21655979.2022.2026553

**Published:** 2022-03-02

**Authors:** Youyu Wang, Bo Jiang, Wang Luo

**Affiliations:** a Department of Thoracic Surgery, The First Affiliated Hospital of Shenzhen University, Shenzhen Second People’s Hospital, Shenzhen, Guangdong, China; b Department of Thoracic Surgery, The Eighth Affiliated Hospital of Sun Yat-sen University, Shenzhen, Guangdong, China; c Departments of Respiratory Diseases, Zengcheng Branch of Nanfang Hospital, Southern Medical University, Guangzhou, Guangdong, China

**Keywords:** Oxaliplatin, memantine, oxidative stress, mitochondrial dysfunction, neurotoxicity

## Abstract

Oxaliplatin is an effective chemotherapeutic agent for the treatment of malignant tumors. However, severe oxaliplatin-induced neurotoxicity has been well documented. Memantine is a drug for the management of Alzheimer’s Disease (AD) due to its promising neuroprotective properties. We hypothesize that Memantine possesses a beneficial role against chemotherapy-induced neuronal damages. In this study, we established an oxaliplatin-induced neurotoxicity assay model in human SHSY-5Y neuronal cells and investigated the protective effect of Memantine. We showed that Memantine treatment ameliorated oxaliplatin-elevated intracellular production of reactive oxygen species (ROS), lipid product malondialdehyde (MDA), and NOX-2 expression. Memantine alleviated impairment of the mitochondrial membrane potential and ATP production by oxaliplatin. As a result, Memantine showed a protective role against oxaliplatin-induced cytotoxicity. Moreover, the terminal deoxynucleotidyl Transferase-mediated dUTP nick end labeling (TUNEL) apoptosis assay revealed that Memantine protected oxaliplatin-induced apoptosis through mitigating the ratio of Bax/Bcl-2 and Caspase-3 cleavage. We concluded Memantine ameliorated the neurotoxicity of oxaliplatin in a mitochondrial-dependent pathway.

## Introduction

**Figure 1.**
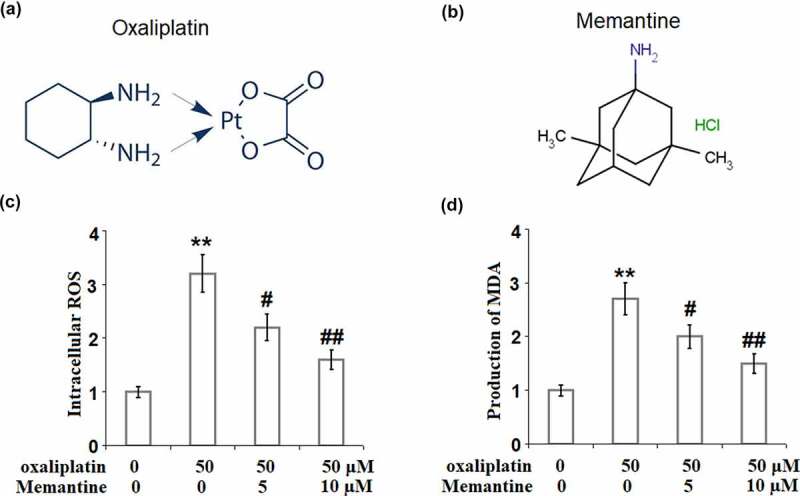
Memantine mitigated oxaliplatin-induced oxidative stress in human SHSY-5Y neuronal cells. Cells were treated with oxaliplatin at 50 μM and Memantine at 5 and 10 µM for 24 hours. (a). The molecular structure of oxaliplatin; (b). The molecular structure of Memantine. (c). Intracellular ROS, the representative images of each condition was illustrated in the upper panel, the quantitative plot was shown in the lower panel; (d). Production of MDA (n = 5–6, **, P < 0.01 vs. vehicle group; #, ##, P < 0.05, 0.01 vs. oxaliplatin group).

Oxaliplatin is a third-generation platinum-based antitumor drug derived from cisplatin and carboplatin and exerts its anti-tumor property through alkylation. The molecular structure of oxaliplatin is shown in Figure 1(a). A broader anticancer spectrum is observed in oxaliplatin than in cisplatin and carboplatin with no cross-drug resistance. Oxaliplatin has been prescribed as one of the basic drugs in chemotherapy for the treatment of advanced ovarian cancer, malignant melanoma, testicular tumor, and lymphoma [1,2]. However, similar to other chemotherapeutic drugs, significant side effects have been reported during clinical treatments, including nausea, vomiting, myelosuppression, peripheral neurotoxicity, and oxaliplatin-induced peripheral neuropathy (OXIN). Moderate to severe neuropathy is observed in approximately 50% of patients after being treated with oxaliplatin for 5–7 months in a dose-dependent manner, limiting its dosage and clinical efficacy [3]. Currently, the pathological mechanism underlying OXIN is unclear. It has been recently reported that mitochondrial-dependent oxidative stress is involved in the development of the neurotoxicity of oxaliplatin [4,5]. The N7 atom on the guanine or adenine in the DNA molecule is the target of oxaliplatin, contributing to the production of 1, 2-d(GDG). 1, 2-d(GDG) further interacts with DNA to produce a Pt-DNA complex [6], blocking the replication of DNA at the G2-M phase [7]. Consequently, the apoptotic signaling pathways, such as PARP and p53 are activated to induce the apoptosis of neurons. In addition, similar changes are observed in mitochondrial DNA. Compared to nuclear DNA, mitochondrial DNA is relatively unstable due to a lack of surrounding histones and the complicated morphology of chromatin, making it easier for oxaliplatin to bind with mitochondrial DNA [8]. The mutated mitochondrial DNA results in the dysfunction of the respiratory chain. Typically, the electrons react with reduced hydrogen and oxygen in the respiratory chain to produce H_2_O with an approximately 3% possibility of leakage [9]. However, when the respiratory chain is damaged, the possibility of electron leakage is greatly promoted and the transudatory electrons bind with oxygen to produce superoxide anions, which are the origin of reactive oxygen species (ROS) [10]. When the balance between the production and the elimination of ROS is broken, oxidative stress is induced by the accumulated ROS, further contributing to neuronal damage [11]. Therefore, preventing oxidative stress by inhibiting ROS generation, might be an effective way to reverse OXIN. NADPH oxidases family (NOX) is another important source of ROS production. Among the NOX family, NOX-2 is mainly responsible for the neuronal damage and the subsequent inflammatory response in neurotoxicity studies [12]. A recent study confirmed the critical role of NOX-2 in the OXIN model [13].

Memantine was approved by China Food and Drug Administration (CFDA) in 2006 for the treatment of Alzheimer’s Disease (AD) [14–16]. The molecular structure of Memantine is shown in Figure 1(b). It is widely reported that Memantine significantly alleviates the declined cognitive function, abnormal behavior, and overall pathological changes in severe AD patients, accompanied by a decline in the total cost of health and social services [17]. Recently, it has been reported that Memantine has a significant beneficial effect on injured neurons by ameliorating inflammation and oxidative stress [17,18]. This evidence supports that Memantine is a promising neuroprotective agent. We hypothesized that Memantine could play a beneficial role against chemotherapy-induced neuropathy. In this study, we established an oxaliplatin-induced neurotoxic model in neuronal cells, and investigated the protective effect of Memantine against OXIN and uncovered the underlying mechanism.

## Materials and Methods

### Cells and treatments

Human SHSY-5Y neuronal cells were purchased from the Type Culture Collection of the Chinese Academy of Sciences (TCCCAS, Shanghai, China) and cultured in the DMEM completed medium supplemented with 10% FBS and CO_2_ at 37°C. The cells were recovered in a 150 mm cell culture dish and split by trypsinization until 90% confluence was reached. For the experiment, 2 × 10^5^ cells /well were cultured in a 6-well plate and treated with 50 μM oxaliplatin (Cat#HY-17371, MedChemExpress, USA) [4,5] in the presence or absence of Memantine (Cat#1189713-18-5, Chemgen bio-Tech Pioneer, USA) [17] at the concentrations of 5 or 10 µM for 24 hours. In this study, cells from passages 3 to 6 were used for experiments.

### DCFH-DA staining

SHSY-5Y neuronal cells were planted on the wells at a density of 1× 10^5^ cells/well and treated for 24 hours. To measure cellular ROS production, the cells were mixed with 1 mL DCFH-DA (Sigma-Aldrich, Missouri, USA) solution after removing the culture medium. The DCFH-DA solution was diluted using the serum-free medium at a ratio of 1:1000. After incubation for 20 minutes at 37°C, the cells were washed with PBS buffer to remove the residual DCFH-DA solution. Finally, the cells were visualized with an inverted fluorescence microscope (Olympus, Tokyo, Japan).

### The measurement of MDA

SHSY-5Y cells were plated on a 6-well plate with a density of 2× 10^5^ cells/well and treated for 24 hours. The concentration of malondialdehyde (MDA) was determined using the thiobarbituric acid (TBA) assay according to the method described previously [19].

### Real-time PCR analysis

The total RNA was isolated from the treated SHSY-5Y neuronal cells on a 6-well plate using a commercial kit (Sigma, Massachusetts, USA). It was further reversely transcribed into cDNA using the cDNA Synthesis kit (Bio-Rad, USA). The TaqMan system (Thermo, Massachusetts, USA) was used to perform the real-time PCR. GAPDH was used to normalize the relative expression of target genes, and calculated with the 2^−ΔΔCt^ method. The following primer sequences were used: NOX-2, forward: 5’-ATACTCGAGCTTGTCTCTTCCATGAGGAAATAAATG-3’; Reverse: 5’-ATTAATTAGCGGCCGCGAAAGCTCATTCATTTTAATAG-3’. GAPDH, Forward: 5’-CCTCGTCCCGTAGACAAAATG-3’; Reverse: 5’-TGAGGTCAATGAAGGGGTCGT-3’.

### Western blot assay

The lysis buffer (Beyotime, China) was used to isolate the protein from the treated SHSY-5Y neuronal cells at a density of 5× 10^5^ cells per culture dish, then quantified using a BCA kit (Beyotime, China). Subsequently, 20 μg of protein were loaded and separated with the SDS-PAGE and further transferred to the PVDF membrane (Beyotime, Shanghai, China). Then, 5% BSA solution was used to incubate the membrane to clear the nonspecific binding proteins and the membrane was incubated with the primary antibodies against Bax, Bcl-2, cytochrome C, cleaved caspase 3, or β-actin, respectively. After being washed with TBST buffer, the ECL solution was used to develop the blot. The bands were analyzed utilizing the Image J software (National Institutes of Health, USA) for densitometric analysis.

### Rhodamine 123 staining

Rhodamine 123 staining (Beyotime, China) assay was used to measure the mitochondrial membrane potential (ΔΨm) in the treated SHSY-5Y neuronal cells in a 6-well plate as described above. Briefly, the neuronal cells were planted on a 24-well plate. Then, 2 μM Rhodamine 123 was used to stain the cells for 15 minutes, which were then washed three times using the PBS buffer. Lastly, the fluorescence microscope (Olympus, Tokyo, Japan) was used to take the image of the cells and the decrease in Rhodamine 123 fluorescence indexed the dissipated ΔΨm. The intensity of Rhodamine 123 staining was assessed using the software Image J (NIH, USA).

### ATP determination

A commercial ATP kit (Roche, Germany) was used to determine the ATP concentration in the treated SHSY-5Y neuronal cells in a 6-well plate as described above. Briefly, the cells were treated with the lysis buffer and thereafter centrifuged to obtain the supernatant. Then the supernatant was mixed with the reaction buffer at a ratio of 1:10, measured using the GLOMAX luminometer (Promega Co., Madison, WI, USA). Finally, the concentration of the ATP was determined based on the ATP standard curve according to the manufacturer’s instructions.

### MTT assay

The treated SHSY-5Y neuronal cells were cultured in a 96-well plate at a density of 1× 10^5^ cells/well for 24 hours, and their medium was placed with MTT (0.5 mg/ml) solution to be incubated for 3 hours at 37°C. Subsequently, the medium was replaced with the DMSO solution to dissolve the formazan crystals. After being incubated for 10 minutes, the ELISA microplate reader (Thermo, USA) was used to determine the OD value at 540 nm.

### Release of LDH

The LDH assay kit (Beyotime, Shanghai, China) was used to detect the release of LDH to evaluate the cellular membrane permeability. Briefly, the cells were planted on the 96-well plate, and then LDH-working reagent was added for 30 minutes. The absorbance at 490 nm was measured using a spectrophotometer (Thermo, Massachusetts, USA).

### TUNEL assay

The apoptosis of the treated SHSY-5Y neuronal cells was evaluated with the TUNEL assay using an in-situ Cell Death Fluorescein Detection Kit (Roche, Basel, Swiss) according to the manufacturer’s instructions. In brief, the cells were washed and fixed with 4% paraformaldehyde for 15 minutes, then subsequently incubated with the reaction solution for 30 minutes at 37°C in the dark. Then, the cells were counted and analyzed using a confocal laser scanning fluorescence microscope (Olympus, Tokyo, Japan). Finally, an image analyzing system (Leica Qwin, Cambridge, UK) was used to detect the percentage of stain-positive cells.

### Isolation of mitochondria and cytosol

The mitochondria and cytosol of the treated SHSY-5Y neuronal cells at a density of 5× 10^5^ cells per culture dish were separated using differential centrifugation to isolate cytosolic and mitochondrial fractions as previously described [20].

### Statistical analysis

The results are presented as mean ± standard deviation (S.D.). Statistical significance was assessed using ANOVA with Tukey’s post-hoc test. A p-value of < 0.05 was considered statistically significant.

## Results

This study established a cytotoxicity assay model in human SHSY-5Y neuronal cells. We treated the cells with the chemotherapy drug oxaliplatin in the presence of Memantine. Our data show that the addition of Memantine significantly ameliorated oxaliplatin-induced ROS production and mitochondrial dysfunction. As a result, the presence of Memantine showed a protective effect against oxaliplatin-induced cytotoxicity and apoptosis.

### Memantine mitigated oxaliplatin-induced oxidative stress in human SHSY-5Y neuronal cells

To explore the effect of Memantine on oxidative stress induced by oxaliplatin, the ROS level, and the MDA concentration were detected after the cells were treated with oxaliplatin at 50 μM and Memantine at two doses (5, and 10 µM) for 24 hours. An obvious upregulation of the ROS level was found in the SHSY-5Y neuronal cells stimulated with oxaliplatin (Figure 1(c)), which was significantly suppressed by Memantine. In addition, the promoted concentration of MDA (Figure 1(d)) induced by oxaliplatin was greatly suppressed by treatment with Memantine. These results demonstrate that oxidative stress in the neuronal cells induced by oxaliplatin was obviously reversed by Memantine.

### Memantine prevented oxaliplatin-induced expression of NOX-2

**Figure 2.**
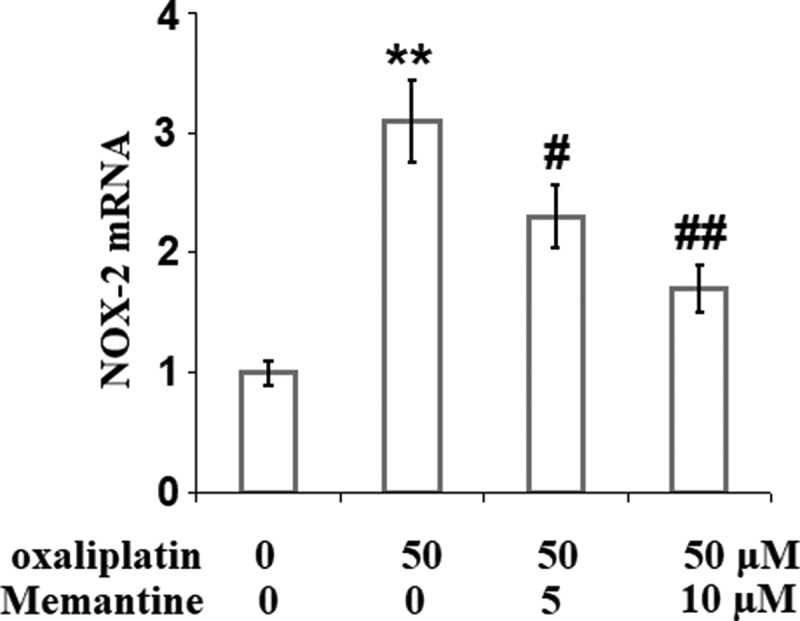
Memantine prevented oxaliplatin-induced expression of NOX-2 in human SHSY-5Y neuronal cells. Cells were treated with oxaliplatin at 50 μM and Memantine at 5 and 10 µM for 24 hours. mRNA of NOX-2 as measured by real-time PCR (n = 6,**, P < 0.01 vs. vehicle group; #, ##, P < 0.05, 0.01 vs. oxaliplatin group).

To explore the potential target protein responsible for the protective effect of Memantine on the increased oxidative stress, the expression level of NADPH oxidase-2 (NOX-2) was detected. As shown in Figure 2, NOX-2 was found to be remarkably increased by stimulation with oxaliplatin but was greatly reduced by the introduction of Memantine, indicating that NOX-2 might be a specific target of Memantine.

### Memantine ameliorated oxaliplatin-induced mitochondrial dysfunction

**Figure 3.**
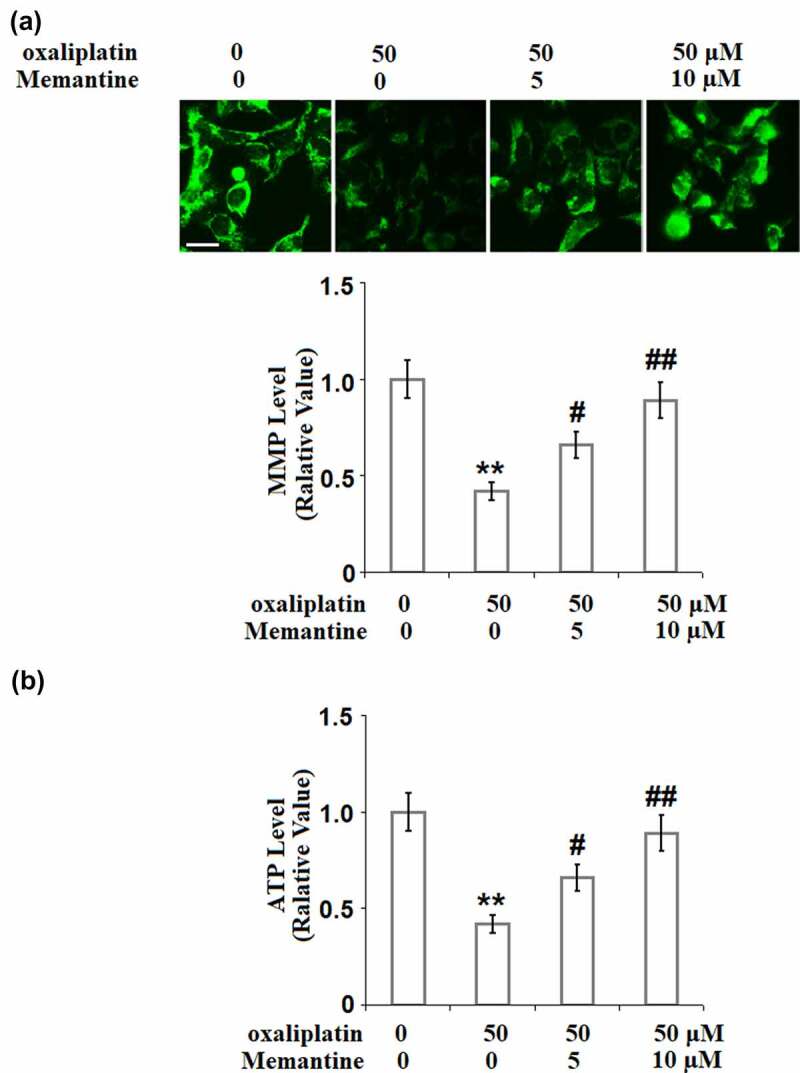
Memantine ameliorated oxaliplatin-induced mitochondrial dysfunction in SHSY-5Y neuronal cells. Cells were treated with oxaliplatin at 50 μM and Memantine at 5 and 10 µM for 24 hours. (a). The change of Mitochondrial membrane potential (ΔΨm), the representative images of each condition was illustrated in the upper panel, the quantitative plot was shown in the lower panel; (b). Production of ATP (n = 5**, P < 0.01 vs. vehicle group; #, ##, P < 0.05, 0.01 vs. oxaliplatin group).

To further evaluate the protective effect of Memantine on the mitochondrial dysfunction induced by oxaliplatin, the mitochondrial membrane potential and the production of ATP were checked. As shown in Figure 3(a), the mitochondrial membrane potential in the SH5Y neuronal cells was significantly suppressed by stimulation with oxaliplatin but was significantly elevated by Memantine. In addition, as shown in Figure 3(b), the suppressed ATP production in the cells induced by oxaliplatin was greatly promoted by Memantine. These results demonstrate that Memantine partly attenuated the mitochondrial dysfunction in the neurons induced by oxaliplatin.

### Memantine attenuated oxaliplatin-induced reduction of cell viability and release of LDH in human SHSY-5Y neuronal cells

**Figure 4.**
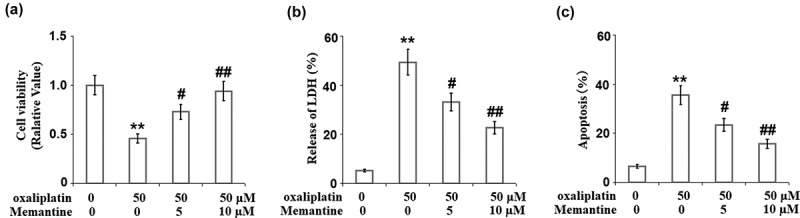
Memantine ameliorated oxaliplatin-induced reduction of cell viability in human SHSY-5Y neuronal cells. Cells were treated with oxaliplatin at 50 μM and Memantine at 5 and 10 µM for 24 hours. (a). Cell viability; (b). Release of LDH. (c) Apoptosis result (n = 6,**, P < 0.01 vs. vehicle group; #, ##, P < 0.05, 0.01 vs. oxaliplatin group).

We further evaluated the effect of Memantine on the proliferation ability of the cells in the presence of oxaliplatin. The suppressed cell viability (Figure 4(a)) induced by oxaliplatin was dose-responsively rescued by treatment with Memantine. In addition, compared to the control, the release of LDH in the oxaliplatin-treated cells was elevated from 5.3% to 49.3% but was suppressed to 33.1% and 22.7% in the 5 and 10 µM Memantine-treated neuron cells, respectively (Figure 4(b)).

### Memantine attenuated oxaliplatin-induced apoptosis in human SHSY-5Y neuronal cells

The apoptosis of neuronal cells was determined using a TUNEL assay. Compared to the control, the apoptotic rate of oxaliplatin-treated cells was increased from 6.6% to 35.6% and was suppressed to 23.4% and 15.7% in the 5 and 10 µM Memantine-treated neuronal cells (Figure 4(c)), respectively, indicating that the apoptosis in the neuronal cells induced by oxaliplatin was significantly alleviated by treatment with Memantine.

### Memantine mitigated oxaliplatin-induced alteration in the ratio of Bax/Bcl-2

**Figure 5.**
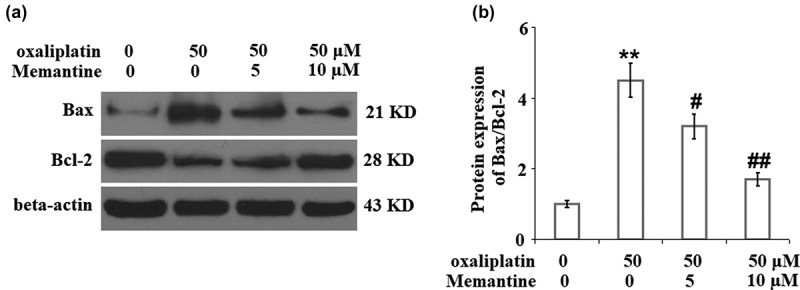
Memantine mitigated oxaliplatin-induced alteration in the ratio of Bax/Bcl-2. Cells were treated with oxaliplatin at 50 μM and Memantine at 5 and 10 µM for 24 hours. (a). Protein expression of Bax and Bcl-2; (b). Statistical analysis of Bax/Bcl-2 (n = 5, **, P < 0.01 vs. vehicle group; #, ##, P < 0.05, 0.01 vs. oxaliplatin group).

As shown in Figure 5(a,b), the elevated expression of Bax and decreased expression of Bcl-2 in the neuron cells induced by stimulation with oxaliplatin were significantly reversed by treatment with Memantine.

### Memantine mitigated oxaliplatin-induced release of cytochrome C from mitochondria to cytosol and the cleavage of caspase-3

**Figure 6.**
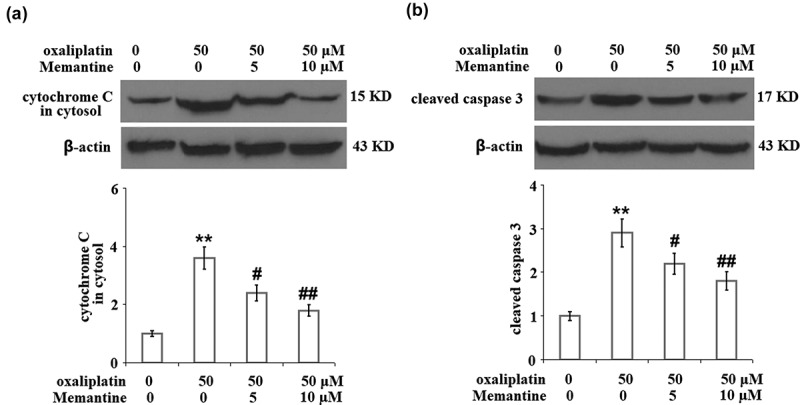
Memantine mitigated oxaliplatin-induced release of cytochrome C from mitochondria to cytosol and the cleavage of caspase 3 in total lysate. Cells were treated with oxaliplatin at 50 μM and Memantine at 5 and 10 µM for 24 hours. (a). The expression of cytochrome C in cytosol; (b). The level of cleaved and total caspase 3, the representative plots was illustrated in the upper panel, the quantitative plot and statistical analysis was shown in the lower panel (n = 5,**, P < 0.01 vs. vehicle group; #, ##, P < 0.05, 0.01 vs. oxaliplatin group).

As shown in Figure 6(a), the expression of cytochrome C in the isolated cytosol was obviously increased by stimulation with oxaliplatin but was greatly suppressed by the introduction of Memantine. In addition, the upregulated expression level of cleaved caspase-3 in the oxaliplatin group was remarkably downregulated by treatment with Memantine (Figure 6(b)).

## Discussion

Oxaliplatin-induced impairment of the respiratory chain in the neurons is one of the main inducers of neurotoxicity, which contributes to the significant decrease in the production of ATP and the mitochondrial membrane potential [21]. As the mitochondrial permeability transition pore (mPTP) opens, some negatively charged soluble proteins are released into the cytoplasm to maintain the mitochondrial transmembrane potential, which further decreases the mitochondrial membrane potential. The loss of multiple soluble proteins and the oligomerization of the apoptotic factors, such as Bax and Bak, subsequently resulted in their transport from the cytoplasm to the outer mitochondrial membrane and interaction with the voltage-dependent anion channel on the membrane. Eventually, mPTP is opened nonspecifically and the substances located in the mitochondria are released into the cytoplasm unrestrictedly, including the cytochrome C, AIF, caspase, endonuclease G (Endo G), and RNAs expressed in the mitochondria [22]. Apoptosis or protein variation are both induced when these factors are released into the cytoplasm [23]. Consequently, the toxicity leads to the damage of the neuron cells. In the present study, mitochondrial dysfunction in SHSY-5Y neuronal cells was observed after the stimulation of oxaliplatin, which was verified by the decreased mitochondrial transmembrane potential and production of ATP. After treating the injured SHSY-5Y neuronal cells with two doses of Memantine, we found that the mitochondrial dysfunction was significantly alleviated, indicating a potential protective effect of Memantine against oxaliplatin-induced mitochondrial injury in the neuron cells. Also, we found that oxaliplatin suppressed neuronal proliferation but induced apoptosis, which was revealed by the decreased cell viability, increased LDH release and enhanced TUNEL staining. The expressions of pro-apoptotic factors, including Bax [24], cytochrome C [25], and cleaved caspase-3 [26], were found to be significantly elevated, and the expression of anti-apoptotic factor, Bcl-2 [27], was found to be downregulated by oxaliplatin, suggesting its apoptosis-inducing property. The treatments with Memantine significantly reversed the suppressed proliferation rate, induced apoptosis, and the alteration of Bax, Bcl-2, cytochrome C, and cleaved caspase-3, indicating Memantine possesses an anti-apoptotic property against oxaliplatin-induced injury to neuron cells. In our future study, the in-vivo neuroprotective effect of Memantine will be tested on the animal nerve injury model induced by the administration of oxaliplatin to provide more evidence for the clinical application of Memantine.

Excessive accumulation of ROS caused by mitochondrial dysfunction results in extensive damage inside the nerves [28]. The release of pro-inflammatory factors and growth factors from the neuroglial cells are induced by oxidative stress, increasing the sensitivity of the damaged area in the peripheral nerve. Several oxidation reactions are induced by oxidative stress, including oxidation of phospholipid to destroy myelin, oxidation of proteins to activate the transient receptor potential vanilloid (TRPV) channel, oxidation of anti-oxidase to aggravate the accumulation of ROS, and oxidation of DNA to destroy DNA structure [29,30]. Therefore, severe injury is induced in the neurons. Here, oxidative stress was obviously activated by stimulation with oxaliplatin, which was verified by the elevated ROS level, production of MDA, and upregulation of NOX-2. After treatment with Memantine, ROS level and the production of MDA were suppressed and NOX-2 was downregulated, indicating an inhibitory effect of memantine against oxidative stress in the neurons induced by oxaliplatin. In our future work, the potential underlying mechanism, such as the effect of Memantine against the Nrf2 signaling pathway, will be further investigated to better understand the regulatory property of Memantine against oxidative stress.

## Conclusion

In summary, we conclude that the Alzheimer’s drug Memantine has a beneficial effect against the neurotoxicity of oxaliplatin via a mitochondrial-dependent pathway in cultured neuronal cells. The therapeutic effect of Memantine remains to be validated in *in vivo* studies.
